# The data-hypothesis relationship

**DOI:** 10.1186/s13059-021-02276-4

**Published:** 2021-02-10

**Authors:** Teppo Felin, Jan Koenderink, Joachim I. Krueger, Denis Noble, George F.R. Ellis

**Affiliations:** 1 Saïd Business School, University of Oxford, Oxford, UK; 2 Department of Physics, Delft University of Technology, Delft, The Netherlands; 3 Department of Experimental Psychology, University of Leuven, Leuven, Belgium; 4 Department of Cognitive, Linguistic and Psychological Sciences, Brown University, Providence, USA; 5 Department of Physiology, Anatomy and Genetics, University of Oxford, Oxford, UK; 6 Department of Mathematics, University of Cape Town, Cape Town, South Africa

Every conscious cognitive process will show itself to be steeped in theories; full of hypotheses.

Rupert Riedl [1]

In a provocative editorial, Yanai and Lercher (henceforth Y&L) claim that “a hypothesis is a liability” [2]. They contend that having a hypothesis is costly because it causes scientists to miss hidden data and interesting phenomena. Y&L advocate “hypothesis-free” data exploration, which they argue can yield significant scientific discoveries.

We disagree. While we concur that a *bad* hypothesis is a liability, there is no such thing as hypothesis-free data exploration. Observation and data are always hypothesis- or theory-laden. Data is meaningless without some form of hypothesis or theory. Any exploration of data, however informal, is necessarily guided by some form of expectations. Even informal hunches or conjectures are types of proto-hypothesis. Furthermore, seemingly hypothesis-free statistical tools and computational techniques also contain latent hypotheses and theories about what is important—what might be interesting, worth measuring or paying attention to. Thus, while Y&L argue that a “hypothesis is a liability,” we argue that hypothesis-free observation is not possible (nor desirable) and that hypotheses in fact are the primary engine of scientific creativity and discovery.

## The hidden gorilla

To illustrate their point about how a hypothesis is a liability, Y&L present their own version of the famous gorilla experiment [3]. In their experiment, subjects receive some made-up data featuring three variables: the BMI of individuals, the number of steps taken on a particular day, and their gender. One experimental group received three hypotheses to consider, while the other was “hypothesis-free.” Subjects in this latter group were simply asked to address the question “what do you conclude from the dataset?”

The “catch” of Y&L’s experiment was that a visual plot of the data showed a waving gorilla. And the key finding was that subjects in the hypothesis-free group were five times more likely to see the gorilla, compared with subjects in the hypothesis-focused group. Y&L concluded from this that hypotheses blind us to hidden patterns and insights in the data. Perhaps ironically, Y&L come to this conclusion based on their own hypothesis about the dangers of hypotheses.

But how exactly does missing the gorilla generalize to Y&L’s point about a hypothesis being a liability in scientific discovery? They argue that missing the gorilla is a problem, even though it is hard to see how finding an irrelevant gorilla mimics making a scientific insight. Now, we understand the gorilla is used as a metaphor for missing surprising or hidden things in science. But a meteorologist missing a cloud that looks like a gorilla is roughly equivalent to what Y&L are doing. A gorilla-shaped cloud has no scientific interest to the meteorologist, just as the gorilla-shaped data is irrelevant to Y&L’s context (the health data with three variables: BMI, steps taken and gender). Furthermore, the gorilla example does not generalize to scientific discovery because a gorilla is something that is universally recognized, while scientific discovery is essentially about finding new data, establishing new facts and relationships. New insights and scientific discoveries do not somehow “pop out” like the gorilla does once one plots the raw data. Hypotheses are needed. Thus, there is a mismatch between the experiment and what Y&L are claiming, on a number of levels.

Y&L import some of these problems from the original gorilla study [4]. The most serious concern is that various versions of the gorilla study can be seen as a form of attentional misdirection, similar to what is practiced by magicians. Experimental tasks are artificially constructed and designed to prove a specific hypothesis: that people are blind and miss large objects in their visual scenes. Experimenters first hide something in the visual scene, then distract their subjects with other tasks (whether counting basketball passes or asking them to analyze specific hypotheses), and then, voilà, reveal to them what they have missed. The problem is that—whether in science or in everyday life—an indefinite number of things remain undetected when we interact with data or visual scenes. It is not obvious what an apple falling means, without the right question, hypothesis, or theory. Visual scenes and data teem with possibilities, uses and meanings. Of course, the excitement generated by these studies comes from the fact that something so large and surprising—like a gorilla—goes undetected, even though it should be plainly obvious.

But there are deeper issues here. Reductionist forms of science assume that cues and data (somehow) jump out and tell us why they are relevant and important, based on the characteristics of the data itself (the physical properties of the world). In vision science, this assumption is based on research in psychophysics (and inverse optics and ideal observer theory) that focuses on salience as a function of cue or stimulus characteristics. From this perspective, cues and stimuli become data, information, and evidence due to their *inherent* nature [5].

To illustrate the problem with this, consider two stimulus or cue characteristics that are important to various versions of the gorilla study—and central to psychophysics and the cognitive sciences more generally—namely “size” and “surprisingness” [6]. The idea in psychophysics is that these characteristics should make cues salient. For example, researchers embedded an image of a gorilla in the CT scan images of patients’ lungs. They then asked expert radiologists to look for nodules as part of lung-cancer screening. Eighty-three percent of the radiologists missed the gorilla embedded in the image, despite the fact that the gorilla was 48 times the size of the nodules they were looking for [7].

But if radiologists or experimental subjects were asked to, say, “look for something unusual” or to “see if you can find the animal,” they would presumably find the gorilla. Thus, visual awareness or recognition has little to do with size or surprisingness. It has more to do with the question posed by the experimenter or the expectations of experimental subjects. In fact, experimental subjects themselves might suspect that the study actually is not about counting basketball passes or about analyzing health data or finding cancerous nodules in lungs. If subjects think that they are being tricked by experimenters—as is often the case—they might ignore the distracting tasks and priming questions and look for and find the gorilla. Note, again, that the a priori hypothesis of experimenters themselves is that people are blind, and so the experiments themselves are designed to prove this point. Alert subjects might suspect that they are being purposefully distracted and thus try to guess what they are meant to look for and find it.

The key point here is that the “transformation” of raw cues or data to information and evidence is not a straightforward process. It requires some form of hypothesis. Cues and data do not automatically tell us what they mean, whether or why they are relevant, or for which hypothesis they might provide evidence. Size is relevant in some situations, but not in others. Cues and data only become information and evidence in response to the questions and queries that we are asking.

## Fishing expeditions require a net

One alternative to having a hypothesis, Y&L argue, is hypothesis-*free* exploration of data or what they call fishing expeditions. Of course, the idea of engaging in a fishing expedition—as Y&L recognize—has highly negative connotations, suggesting haphazard, unscientific, and perhaps even unethical practices. But they make a valid point: more exploratory and imaginative practices are important in science.

But fishing expeditions are hardly hypothesis-free. That is, fishing expeditions—to extend Y&L’s metaphor—require a net or some type of device for catching fish. Data and insights (just like fish) do not jump out and declare their relevance, meaning, or importance. As put by physical chemist Michael Polanyi, “things are not labelled ‘evidence’ in nature” [8]. The relevant data needs to be identified and lured in some fashion. Even the most exploratory process in science features choices and assumptions about what will count as data and evidence and what should be measured (and how). Any look at data—however preliminary it might be—*necessarily* represents some form of proto-hypothesis: a latent expectation, question, or even guess about what might be lurking, about what might potentially be interesting or relevant and how it might be caught.

In short, there’s no systematic way to extract and identify anything hidden without at least some rough idea of what one is looking for. The tools and devices scientists use are the net, sieve, or filter for capturing relevance and meaning. These nets come in vastly different materials and textures, sizes, types of weights, and anchors. Choices also need to be made about where to cast these nets. There are various ways to use and deploy them (trolling, longline, and so forth). Each choice implies a hypothesis. The choice of fishing net implies a hypothesis about what one is looking for and about what one might expect to catch and see as relevant [9].

Now, it might seem like we are stretching the definition of a hypothesis by including expectations, conjectures, and even the statistical and computational tools that are used to generate insights. But we think it is important to recognize that *any* tool—whether cognitive, computational, or statistical—functions like a net, as it already embodies implicit hypotheses about what matters and what does not. Perhaps these are not full-fledged, formal hypotheses in the sense that Y&L discuss. But they certainly are proto-hypotheses that direct awareness and attention toward what should be measured and what counts as data and evidence. A hypothesis is some form of expectation or question about what one is looking for and about what one expects to find. The identification and collection of data necessarily is of the same form, as one cannot collect *all* data about what is going on in the world at a specific time: flu patterns in China, weather patterns in the Pacific, sunspot cycles, the state of the New York stock exchange, earthquakes in Tahiti, and so on. Science is about making decisions about what subset of all this “stuff” should be focused on and included in the analysis.

Y&L specifically emphasize correlations and the generation of various statistical patterns as a way to make hypothesis-free discoveries in data. Correlations are one form of “net” for looking at data. But correlations are ubiquitous and their strength tells us little [10]. One needs a hypothesis to arbitrate between which correlation might be worth investigating and which not. The genome-wide associational studies have pointed this out. With the exception of the usual outliers (rare genetic diseases), the association levels are relatively small. More data may offer more stable statistical estimates, but it will not achieve the identification of causality required for a physiological explanation. On the contrary, the extremely low association data can be hiding substantial causality or perhaps more complex or interconnected, omnigenic factors are at play in the genome [11]. A causal hypothesis, tested rigorously with quantitative modeling, can reveal the potential pathways for understanding genetic variation, epigenetic factors, and disease or traits [12].

## Science: bottom-up versus top-down

Y&L argue that scientific discoveries are “undiscoverable without data.” While this is correct in principle, Y&L mis-specify the data-hypothesis relationship by privileging the role the data to the detriment of hypothesis and theory. They ignore the temporal primacy of theory and hypothesis. A hypothesis tells us what data to look for. Data emerges and becomes evidence in response to a hypothesis. In physics, for example, the existence of gravitational waves had long been hypothesized. The hypothesis guided scientists to look for this data. This specifically led to the invention and construction of exquisitely sensitive devices to detect and measure gravitational radiation (e.g., LIGO and VIRGO observations). Eventually, in 2015, gravitational waves were discovered. The data emerged because of the conceptualization, design, and construction of relevant devices for measurement. The data was manifest due to the hypothesis rather than the other way around. And the data analysis itself is theory-based [13]: it depends on templates of waves expected from the gravitational coalescence of black holes or neutron stars.

**Fig. 1:**
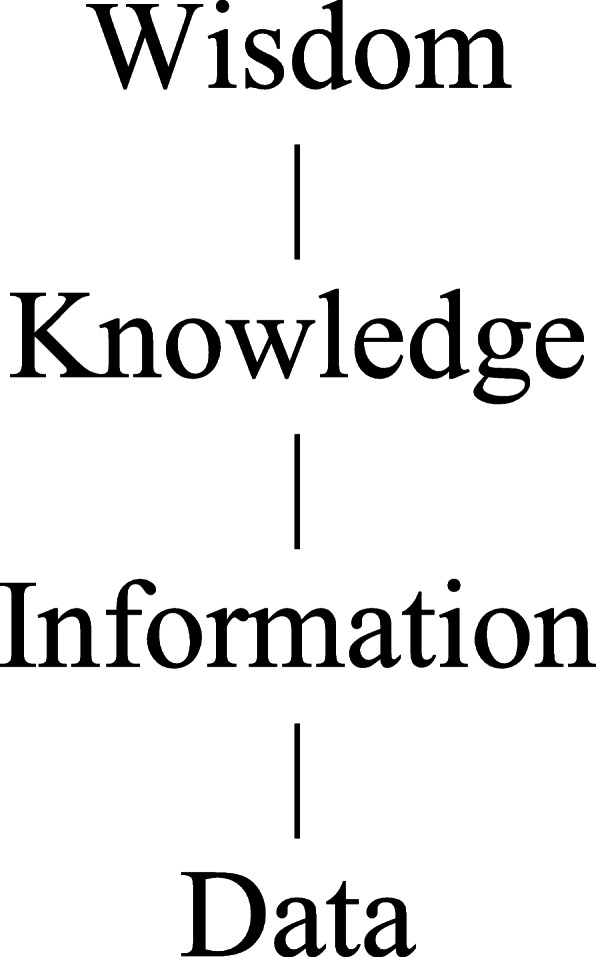
The DIKW “hierarchy” is often seen as “bottom-up.” But, as we argue, top-down mechanisms play a critical role in discovering data, relevance, and meaning

Einstein aptly captured the relationship between hypotheses and data when noting that “whether you can observe a thing or not depends on the theory which you use. It is the theory which decides what can be observed.” Einstein’s point might be illustrated by the so-called DIKW hierarchy (Fig. 1) [14]. Currently popular data-first approaches assume that scientific understanding is built from the bottom-up. But to the contrary, many of the greatest insights have come “top-down,” where scientists start with theories and hypotheses that guide them to identify the right data and evidence. One of the most profound ways this happens is when scientists query fundamental assumptions that are taken for granted, such as that species are fixed for all time, or that simultaneity is independent of the state of motion. This questioning of axiomatic assumptions drives the creation of transformational theories (the theory of evolution, special relativity) and the subsequent collection of associated data that tests such profound reshaping of the foundations.

There certainly are significant reciprocal influences between these “levels” of the hierarchy. But Y&L’s central argument that a “hypothesis is a liability” simply does not recognize the profound, top-down influence played by hypotheses and theories in science, and how these enable the identification and generation of data.

Our concern is that starting at the bottom—as suggested by Y&L’s notion of hypothesis-free exploration of data—will inadvertently lead to an overly descriptive science: what Ernest Rutherford called “stamp collecting.” Charles Darwin anticipated this problem when he wrote to a friend:

It made me laugh to read of [Edwin Lankester’s] advice or rather regret that I had not published *facts alone*. How profoundly ignorant he must be of the very soul of observation. About 30 years ago there was much talk that Geologists ought *only to observe and not theorise*; and I well remember someone saying, that at this rate a man might as well go into a gravel-pit and count the pebbles and describe their colours. How odd it is that everyone should not see that *all observation must be for or against some view*, if it is to be of any service [15].
